# Supplementary material for: Cerium Oxide Nanoparticles Regulate Oxidative Stress in HeLa Cells by Increasing the Aquaporin-Mediated Hydrogen Peroxide Permeability
Source: Int J Mol Sci. 2022 Sep 16;23(18):10837. doi: 10.3390/ijms231810837 (PMC9506032; doi:10.3390/ijms231810837)
Supplement: Supplementary file 1 [file ijms-23-10837-s001.zip › ijms-1852434-supplementary.pdf]

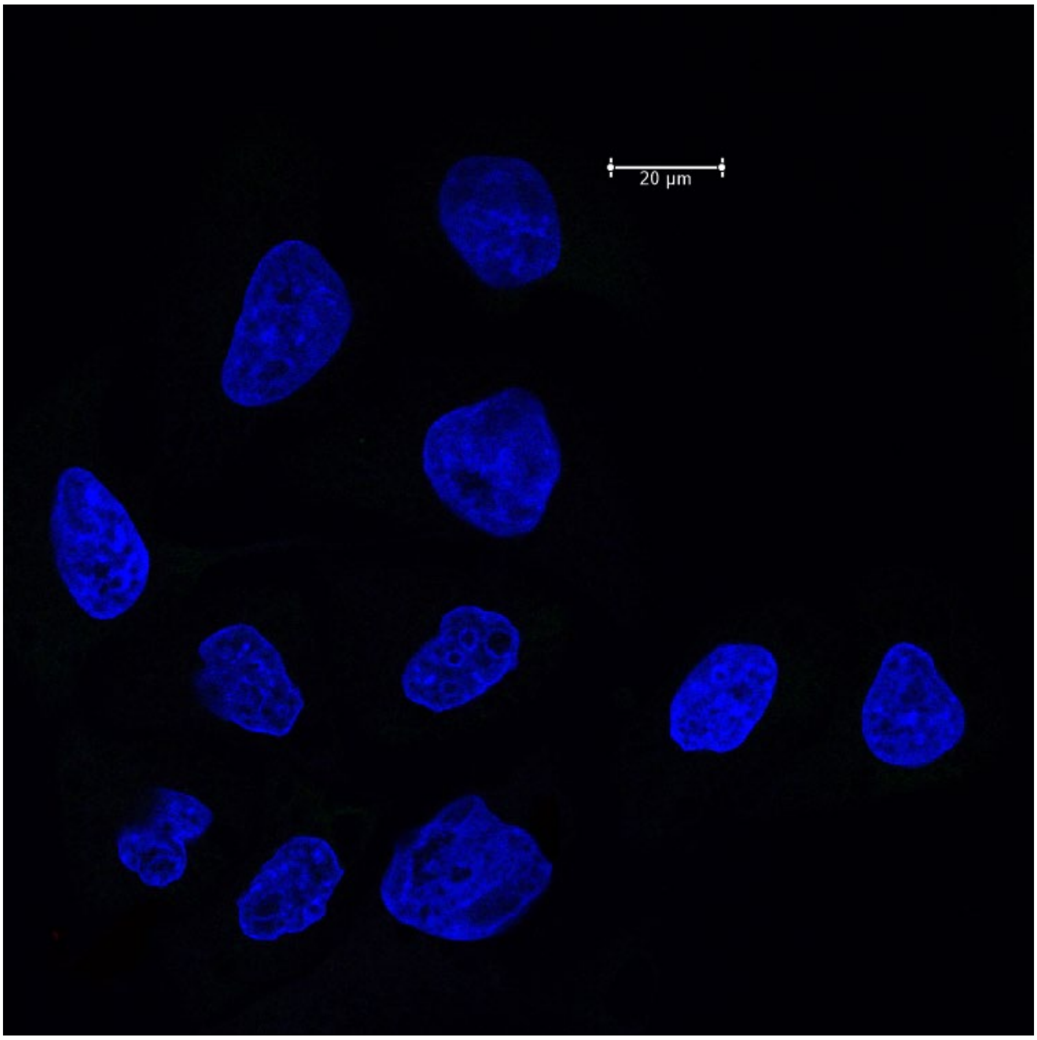

Supplementary Figure S1. Representative immunofluorescence negative control. No or faint staining was observed when anti-aquaporin antibodies and CNPs were substituted with preimmune serum. Nuclei were counterstained by DAPI (blue). Scale bar, 20 μm.

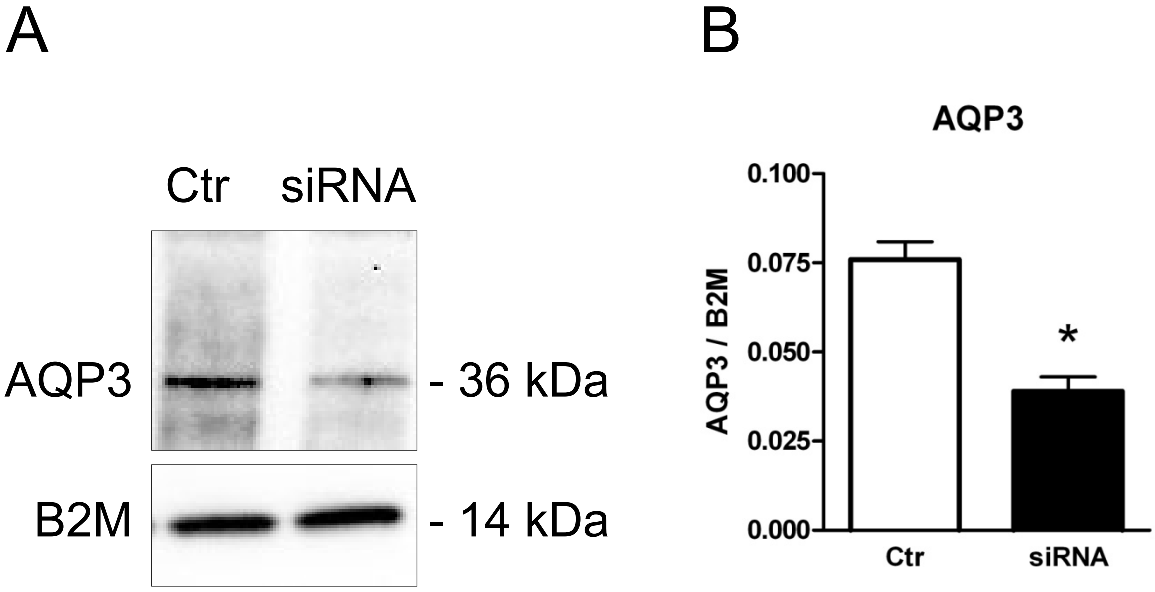

Supplementary Figure S2. AQP3 silencing in HeLa cells. (**A**) Blots representative of three were shown. Lanes were loaded with 30 μg of proteins and probed with anti-AQP3 rabbit polyclonal antibody as described in the Materials and Methods. The same blots were stripped and re-probed with anti-β2microglobulin (B2M) antibody, as housekeeping. (**B**) Densitometry was performed by acquiring the blot with thei BrightCL1000 imaging system. Semiquantitation was performed by iBA (iBright Analysis Software) and the results were normalized to the corresponding B2M.
Densitometry demonstrated a significant reduced protein expression in silenced (siRNA) cells compared to controls (scrambled; Ctr) (*, $p < 0.05$; Student's *t* test).

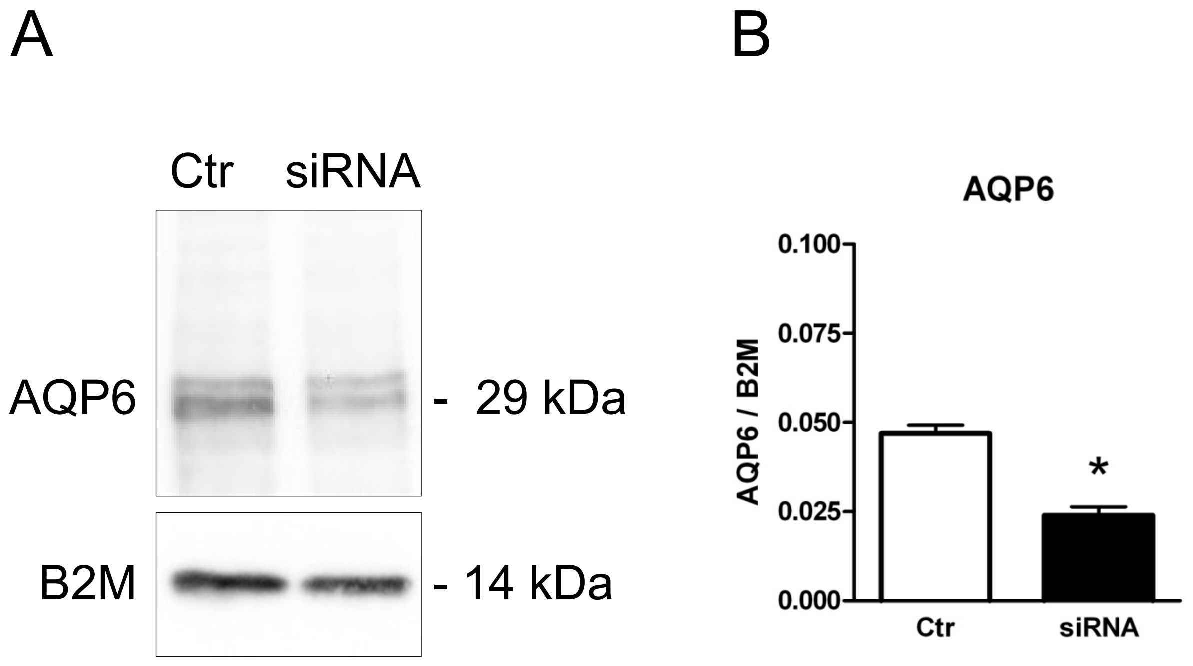

Supplementary Figure S3. AQP6 silencing in HeLa cells. (**A**) Blots representative of three were shown. Lanes were loaded with 30 μg of proteins and probed with anti-AQP6 rabbit polyclonal antibody as described in the Materials and Methods. The same blots were stripped and re-probed with anti-β2microglobulin (B2M) antibody, as housekeeping. (**B**) Densitometry was performed by acquiring the blot with thei BrightCL1000 imaging system. Semiquantitation was performed by iBA (iBright Analysis Software) and the results were normalized to the corresponding B2M.Densitometry demonstrated a significant reduced protein expression in silenced (siRNA) cells compared to controls (scrambled; Ctr) (*, $p < 0.05$; Student's *t* test).

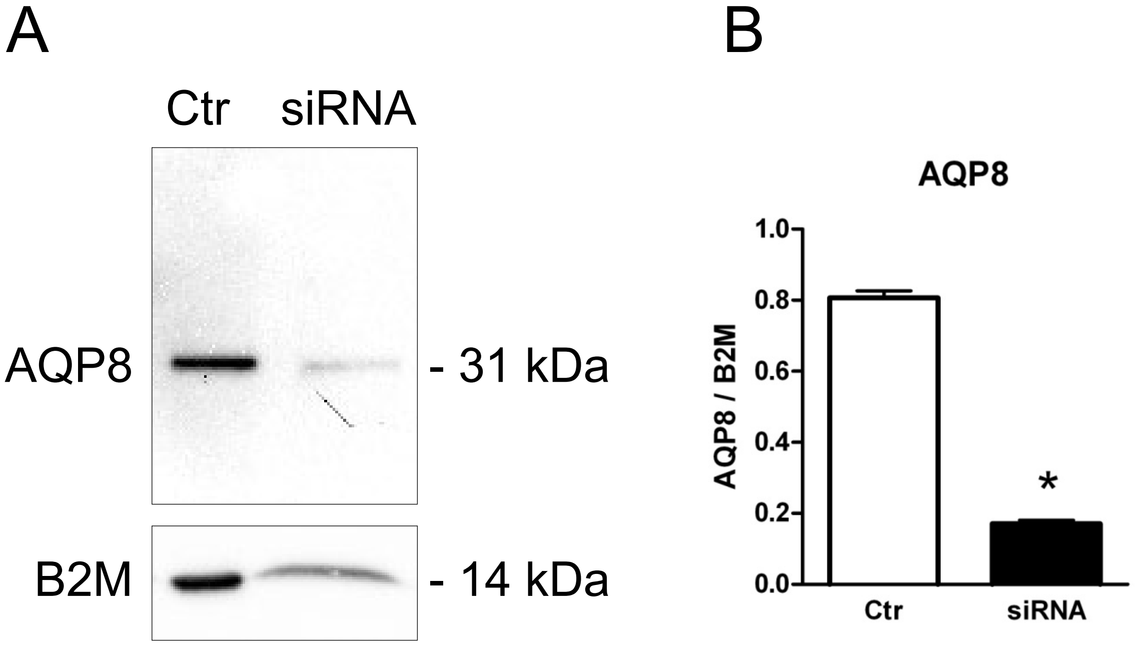

Supplementary Figure S4. AQP8 silencing in HeLa cells. (**A**) Blots representative of three were shown. Lanes were loaded with 30 μg of proteins and probed with anti-AQP8 rabbit polyclonal antibody as described in the Materials and Methods. The same blots were stripped and re-probed with anti-β2microglobulin (B2M) antibody, as housekeeping. (**B**) Densitometry was performed by acquiring the blot with thei BrightCL1000 imaging system. Semiquantitation was performed by iBA (iBright Analysis Software) and the results were normalized to the corresponding B2M.

Densitometry demonstrated a significant reduced protein expression in silenced (siRNA) cells compared to controls (scrambled; Ctr) (*, $p < 0.05$; Student's *t* test).

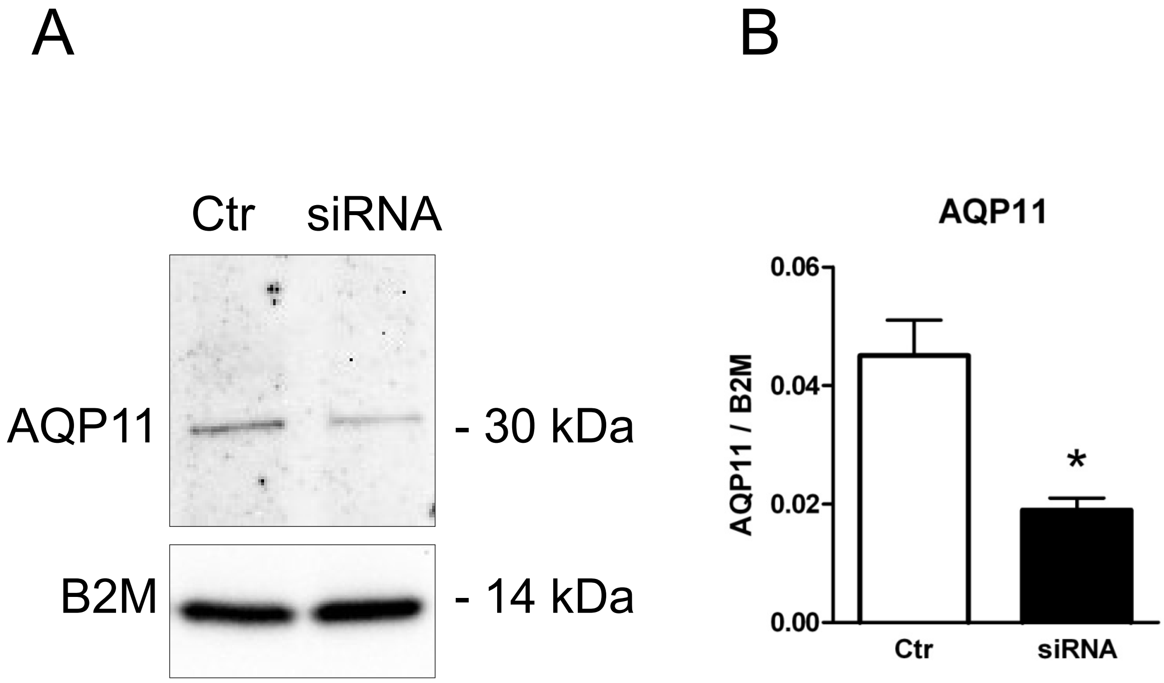

Supplementary Figure S5. AQP11 silencing in HeLa cells. (**A**) Blots representative of three were shown. Lanes were loaded with 30 μg of proteins and probed with anti-AQP11 rabbit polyclonal antibody as described in the Materials and Methods. The same blots were stripped and re-probed with anti-β2microglobulin (B2M) antibody, as housekeeping. (**B**) Densitometry was performed by acquiring the blot with thei BrightCL1000 imaging system. Semiquantitation was performed by iBA (iBright Analysis Software) and the results were normalized to the corresponding B2M.

Densitometry demonstrated a significant reduced protein expression in silenced (siRNA) cells compared to controls (scrambled; Ctr) (*, $p < 0.05$; Student's *t* test).

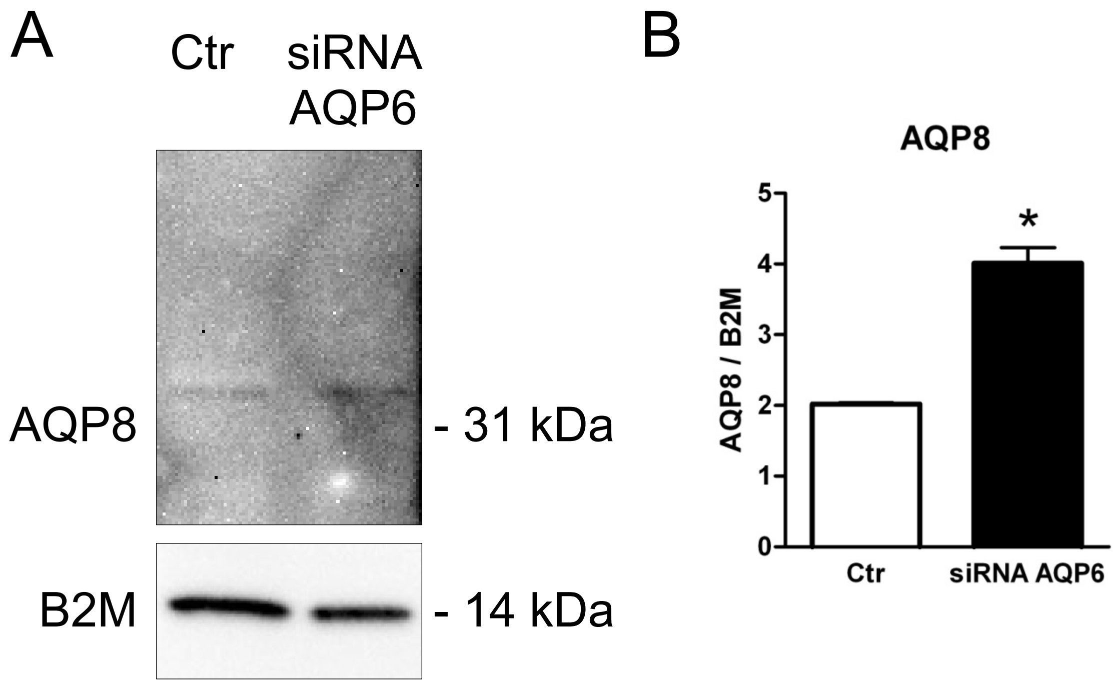

Supplementary Figure S6. Upregulation of AQP8 protein in AQP6 silenced HeLa cells. (**A**) Blots representative of three were shown. Lanes were loaded with 30 μg of proteins and probed with anti-AQP8 rabbit polyclonal antibody as described in the Materials and Methods. The same blots were stripped and re-probed with anti-β2microglobulin (B2M) antibody, as housekeeping. (**B**) Densitometry was performed by acquiring the blot with the BrightCL1000 imaging system. Semiquantitation was performed by iBA (iBright Analysis Software) and the results were normalized to the corresponding B2M. Densitometry demonstrated a significant reduced protein expression in silenced (siRNA) cells compared to controls (scrambled; Ctr) (*, $p < 0.05$; Student's *t* test).
